# Meta-QTL analysis and candidate genes for quality traits, mineral content, and abiotic-related traits in wild emmer

**DOI:** 10.3389/fpls.2024.1305196

**Published:** 2024-03-14

**Authors:** Patricia Cabas-Lühmann, Andrés R. Schwember, Osvin Arriagada, Ilaria Marcotuli, Iván Matus, Christian Alfaro, Agata Gadaleta

**Affiliations:** ^1^ Departamento de Ciencias Vegetales, Facultad de Agronomía y Sistemas Naturales, Pontificia Universidad Católica de Chile, Santiago, Chile; ^2^ Centro de Estudios en Alimentos Procesados (CEAP), Talca, Chile; ^3^ Department of Soil, Plant and Food Sciences, University of Bari Aldo Moro, Bari, Italy; ^4^ Instituto de Investigaciones Agropecuarias (INIA), Centro Regional Quilamapu, Chillán, Chile; ^5^ Instituto de Investigaciones Agropecuarias (INIA), Centro Regional Rayantue, Rancagua, Chile

**Keywords:** meta-QTL analysis, QTL, wild emmer, quality traits, mineral composition, abiotic-related traits, disease-related traits

## Abstract

Wild emmer (*Triticum turgidum* ssp. *dicoccoides*) genotypes were studied for their high-nutritional value and good tolerance to various types of stress; for this reason, several QTL (quantitative trait loci) studies have been conducted to find favorable alleles to be introgressed into modern wheat cultivars. Given the complexity of the QTL nature, their interaction with the environment, and other QTLs, a small number of genotypes have been used in wheat breeding programs. Meta-QTL (MQTL) analysis helps to simplify the existing QTL information, identifying stable genomic regions and possible candidate genes for further allele introgression. The study aimed to identify stable QTL regions across different environmental conditions and genetic backgrounds using the QTL information of the past 14 years for different traits in wild emmer based upon 17 independent studies. A total of 41 traits were classified as quality traits (16), mineral composition traits (11), abiotic-related traits (13), and disease-related traits (1). The analysis revealed 852 QTLs distributed across all 14 chromosomes of wild emmer, with an average of 61 QTLs per chromosome. Quality traits had the highest number of QTLs (35%), followed by mineral content (33%), abiotic-related traits (28%), and disease-related traits (4%). Grain protein content (GPC) and thousand kernel weight (TKW) were associated with most of the QTLs detected. A total of 43 MQTLs were identified, simplifying the information, and reducing the average confidence interval (CI) from 22.6 to 4.78 cM. These MQTLs were associated with multiple traits across different categories. Nine candidate genes were identified for several stable MQTLs, potentially contributing to traits such as quality, mineral content, and abiotic stress resistance. These genes play essential roles in various plant processes, such as carbohydrate metabolism, nitrogen assimilation, cell wall biogenesis, and cell wall extensibility. Overall, this study underscores the importance of considering MQTL analysis in wheat breeding programs, as it identifies stable genomic regions associated with multiple traits, offering potential solutions for improving wheat varieties under diverse environmental conditions.

## 1 Introduction

Due to the domestication process and systematic breeding selection, the genetic variability of bread wheat (*Triticum aestivum* L.) and durum wheat (*Triticum turgidum* L. ssp. *durum*) is relatively small compared to the diversity found in their tetraploid progenitor wild emmer (*Triticum turgidum* ssp. *dicoccoides*; 2n = 4x = 28 chromosomes; AB-genomes) (Abbo et al., 2014; Golan et al., 2015). Historically, yield and yield components have been the main selection criteria in breeding programs for any staple crop, including wheat. Generally, there is a negative correlation among yield components and quality traits (Blanco et al., 2012). In fact, some studies have found that durum and bread wheat decreased over selection time in grain protein content (GPC) (Subira et al., 2014), mineral content (Cakmak et al., 2010), and resistance to abiotic and biotic stressors in favor of higher yields, test weight, and thousand kernel weight (TKW) (Peng et al., 2011). Due to the impacts of climate change and the environmental effect on various traits, it is imperative to incorporate tolerance to environmental stressors and diseases into breeding programs, particularly for crops such as wheat. This necessity extends to the inclusion of crops that are both healthier and more nutritious while also avoiding intensive production practices (Longin et al., 2016).

Because of its nutritional values and tolerance to abiotic and biotic stressors, wild emmer has been used as an alternative crop for the introgression of favorable alleles into modern wheat genotypes, expanding the genetic diversity within ongoing wheat breeding programs (Cakmak et al., 2004; Kuznetsova et al., 2019), particularly in the cultivation of the crop under adverse environmental conditions (Beres et al., 2020). The introgression of specific wild emmer genes into modern wheats has been carried out for the principal traits important for breeders, the food industry, and the consumers (Merchuk-Ovnat et al., 2016a, 2017; Kumar et al., 2020; Liu et al., 2021). Joppa and Cantrell (1990) identified the first QTL (quantitative trait loci) for GPC in wild emmer lines “FA-15-3” and “F-28-8-3” from Israel, called *Gpc-B1* on chromosome 6B. This genetic locus increased the overall GPC to 18%, 2% higher than the GPC in the recurrent durum wheat parent “Langdon” (LDN), which had 16.8% (Joppa et al., 1991). The gene *Gpc-B1* encodes for a NO APICAL MERISTEM-B1 (*NAM-B1*), which increases nutrient remobilization from leaves to grains related to the final GPC and mineral composition. However, it was not functional in modern wheat cultivars (Uauy et al., 2006; Avni et al., 2014). Since the discovery of *Gpc-B1*, several other QTLs have been identified; for example, Peleg et al. (2009a) and Fatiukha et al. (2019) mapped several QTLs associated with plant productivity and drought-adaptive traits in a RIL population derived from a cross between durum wheat (LDN) and wild emmer (“G18-16”). Subsequently, some of these wild QTLs were introgressed from G18-16 into an elite Israeli durum material (“Uzan”) on chromosomes 1B and 2B and bread wheat (“Bar Nir” and “Zahir”) on chromosome 7A via marker-assisted selection. The introgressed QTL improved grain yield, biomass, photosynthetic capacity, and root development across different environments, particularly under drought conditions (Merchuk-Ovnat et al., 2016a, b, 2017).

Several studies have identified QTLs in wild emmer in different mapping populations and environments. Meta-QTL (MQTL) analysis developed by Goffinet and Gerber (2000) is an alternative to (i) facilitate the compilation of information regarding consensus QTLs, narrowing the QTL regions for a particular trait across different environments and genetic backgrounds; (ii) allow the study of many traits at once defining their reliable location and effects across different genetic backgrounds and environments determining the molecular markers valuable for marker-assisted selection (MAS) (Goffinet and Gerber, 2000; Khahani et al., 2021; Soriano et al., 2021); (iii) allow the identification of QTLs that have pleotropic effects by determining regions of the genome (MQTL) that contain QTLs for different traits (Said et al., 2013); (iv) the given MQTL information has been used for candidate genes detection and for future cloning of the genes (Colasuonno et al., 2021; Saini et al., 2021).

Meta-QTL analysis has been successfully performed in bread wheat for yield and related traits (Zhang et al., 2010; Liu et al., 2020; Miao et al., 2022; Saini et al., 2022a), grain quality traits (Tyagi et al., 2015), mineral content (Singh et al., 2022), fusarium head blight (FHB) resistance (Löffler et al., 2009; Venske et al., 2019; Saini et al., 2022b), and abiotic resistance (Xu et al., 2017; Guo et al., 2023). In durum wheat, MQTL has been conducted for yield and related traits (Arriagada et al., 2022); quality traits, abiotic and biotic stressors (Soriano et al., 2021); pasta making quality (Roselló et al., 2018); GPC (Saini et al., 2022c); and ortho-MQTL analysis for quality traits (Marcotuli et al., 2022). To the best of our knowledge, the only MTQL study performed using wild emmer was carried out by Avni et al. (2018) for grain weight. However, no MQTL studies have been conducted using different quality traits, mineral composition, abiotic stressors, or diseases such as FHB in wild emmer. Considering the helpful information that can be obtained from MQTL analysis from wild emmer grown in different environments, the aim of the study was to examine all the existing QTL information published in the last 14 years in different environments regarding agronomical and chemical quality traits, mineral composition, abiotic-related traits, and diseases-related traits to identify MQTL regions and candidate genes in wild emmer that can be further used in modern wheats breeding programs in the world.

## 2 Materials and methods

The MQTL analysis for quality traits, mineral composition traits, abiotic-related traits, and disease-related traits involved three main steps: first, a complete compilation of all QTL data associated with quality, mineral composition, abiotic, and diseases already reported for wild emmer. A list of traits that belongs to each category is reported in **Table 1**. Second, the creation of a consensus map where the QTLs previously collected from the literature were projected. Third, the MQTL identification through MQTL analysis.

**Table 1:** Abbreviations and numbers of QTLs for quality, mineral composition, abiotic, and disease-related traits categories reported in the QTL studies for wild emmer.

Category	Trait	Abbreviation	Number of QTLs
Quality	Grain yield	GY	14
	Harvest index	HI	44
	100 kernel weight	HKW	2
	Kernel length	KL	6
	Kernel number per spike	KNPS	9
	Spike dry matter	SDM	18
	Rachis fragility (% shattering)	SHT	2
	Spike length	SL	21
	Length spikelets internode	SL/TSN	2
	Spikelet number per spike	SNS	15
	Number of spikelets with one fertile floret	STSP	1
	Tiller number	TN	4
	Total dry matter	TDM	6
	Thousand kernel weight	TKW	58
	Vegetative dry matter	VDM	7
	Grain protein content	GPC	89
Mineral content	Grain aluminium concentration	GAIC	2
	Grain calcium concentration	GCaC	25
	Grain copper concentration	GCuC	49
	Grain iron concentration	GFeC	31
	Grain potassium concentration	GKC	13
	Grain magnesium concentration	GMgC	33
	Grain manganese concentration	GMnC	8
	Grain phosphorus concentration	G_P_C	26
	Grain sulfur concentration	GSC	54
	Grain selenium concentration	GSeC	13
	Grain zinc concentration	GZnC	26
Abiotic-related traits	Awn length	AL	7
	Chlorophyll content	CHL	31
	Carbon isotope ratio	CIR	36
	Culm length	CL	28
	Days from heading to maturity	DFHM	26
	Days from planting to heading	DFPH	22
	Flowering date	FD	1
	Flag leaf length	FLL	9
	Flag leaf rolling index	FLRI	42
	Flag leaf width	FLW	22
	Osmotic potential at heading	OPH	16
	Plant height	PH	1
	Spike compactness	SC	2
Disease-related traits	Fusarium head blight	FHB	31

### 2.1 Data collection and consensus genetic map construction

A comprehensive bibliographic search on the Web of Science, Google Scholar, and PubMed was conducted to find the QTL studies associated with quality traits, mineral composition traits, abiotic-related traits, and diseases in wild emmer. At the end of an exhaustive search, a total of 17 independent studies from 2009 to 2022 that included F2 mapping populations, recombinant inbred lines (RILs), recombinant inbred chromosome lines (RICs), and backcross (BC) were used. The selected QTL studies must contain the following:

- a map for the population that includes the wild emmer parental wheat information
- the position of QTL, such as peak position (Pos) and CIs
  iii. logarithm of the odds score (LOD) for each QTL
  iv. phenotypic variance percentage for each QTL (PVE or *r* ^2^)

Any QTL study that did not meet these criteria was not considered in our work. The only disease that met the analysis requirements was FHB; therefore, it was the only trait considered in this category.

From the studies, 41 traits were identified. The number of traits per category was 16 for quality traits, 11 for mineral composition traits, 13 for abiotic-related traits, and one for disease-related traits. The name of each trait per category and the number of QTLs per trait are included in **Table 1**. The studies and the QTLs used are available in **Table 2**. The environmental information per study is in the **Supplementary Table 1**.

**Table 2:** Summary of the QTL studies used for the MQTL analysis for different traits in wild emmer.

Reference	Trait	N QTLs	Pop size	Env	WE #acc	Pop type
Avni et al. (2018)	TKW	7	137	4	“Zavitan”	RILs
Buerstmayr et al. (2012)	FHB, AL, FD, SC	8, 7, 1, 2	134, 129,126	4	“Td161”	BC
Buerstmayr et al. (2013)	FHB	8	103	4	“Mt. Gerizim #36”	BC
Chen et al. (2021)	TN	4	116	4	“WE34021”	RILs
Deblieck et al. (2022)	HI, TKW, SNS, SL	1, 1, 2, 1	150	4	“G18-16”	RILs
Fatiukha et al. (2019)	GPC, TKW	46, 19	208	5	“Y12-3”	RILs
Fatiukha et al. (2020)	G_P_C, GAlC, GCaC, GCuC, GFeC, GKC, GMgC, GPC, GSC, GZnC	11,2,12,31,15,9,24,23,29,12	150	3	“G18-16”	RILs
Fatiukha et al. (2021)	CHL, CIR, CL, DFHM, DFPH, FLL, FLRI, FLW, GY, HI, KNPS, OPH, SDM, SL, TKW, VDM	19, 26, 6, 8,6,9,10,22,6,16,5,4,8,19,20,7	150	2	“G18-16”	RILs
Garvin et al. (2009)	FHB	15	99	3	“Israel A”	RICs
Mo et al. (2021)	SNS	13	121	8	“LM001”	RILs
Peleg et al. (2009a)	G_P_C, GCaC, GCuC, GFeC, GKC, GMgC, GPC, GSC, GZnC	15,12,18,13,4,18,20,25,13	152	3	“G18-16”	RILs
Peleg et al. (2009b)	CHL, CIR, CL, DFHM, DFPH, FLRI, GY, HI, OPH, SDM, TDM	12,10,22,18,16,32,8,23,12,10,6	152	2	“G18-16”	RILs
Peleg et al. (2011)	HI, KNPS, TKW	4,4,11	152	2	“G18-16”	RILs
Thanh et al. (2013)	HKW, PH, SHT, SL/TSN, SL, STSP	2,1,2,2,1,1	144	1	“DCC63”	F2
Velu et al. (2017)	GFeC, GZnC	3,1	105	3	“MM 5/4”	RILs
Yan et al. (2018)	GseC	13	152	3	“G18-16”	RILs
Zhou et al. (2021)	KL	6	121	6	“LM001”	RILs

Env, environment; N, number; Pop, population; WE #acc, wild emmer accessions.

Each QTL was treated as independent, even if some of them were detected in multiple environments or genetic backgrounds. If the CI (95%) for the QTL was not reported, it was calculated using the following equations described by Guo et al. (2006):


$$CI\;\left(cM\right)=163÷\left(N\times R^{2}\right)\text{ for RILs}$$

$$CI\;\left(cM\right)=530÷\left(N\times R^{2}\right)\text{BC and }{\text{F}}_{2}\text{ lines}$$

where *N* is the population size and *R* ^2^ is the proportion of the phenotypic variance explained by the QTL.

The collected genetic maps containing their chromosomes, markers, and positions in cM were integrated individually onto the durum wheat reference map developed by Maccaferri et al. (2015). Maccaferri’s map consisted of 30,144 markers spanning 2,631 cM with 11 markers per cM density. The consensus map was constructed using BioMercator 4.2.3 software (Arcade et al., 2004).

### 2.2 Projection of QTL and meta-QTL analysis

The original QTL data from the 17 studies were projected individually onto the created consensus map by following the homothetic approach described by Chardon et al. (2004). A total of 14 studies were projected, followed by the MQTL analysis that was performed per chromosome using the BioMercator V4.2.3 software (Arcade et al., 2004). If the QTL number per chromosome was higher than 10, the Veyrieras algorithm available in the software was used for the MQTL analysis (Veyrieras et al., 2007). The first part of the Veyrieras algorithm indicated the number of MTQL models generated by different criteria. The criteria were the Akaike Information Criterion (AIC), the corrected AIC (AICc), the modified AIC with factor 3 (AIC3), the Bayesian Information Criterion (BIC), and the Average Weight of Evidence (AWE). The best MQTL model was selected based on the lowest number in at least three of the five criteria (Soriano and Alvaro, 2019). Conversely, if the QTL number per chromosome was ≤ 10, the Goffinet and Gerber approach was performed for the MQTL analysis (Goffinet and Gerber, 2000). The final identification of the number of MQTL per chromosome was based on a delta value ≥ 0.9 value in at least two studies.

### 2.3 Identification of candidate genes

Gene annotations for the most important marker-trait associations (MTAs) were performed using the high-confidence genes reported for the wheat genome sequence (Svevo browser), available at https://wheat.pw.usda.gov/GG3/jbrowse_Durum_Svevo. The marker locations were defined by flanking marker positions and the CI of the MQTL.

## 3 Results

### 3.1 Features for the studied QTLs under diverse growing environments

**Figure 1:**
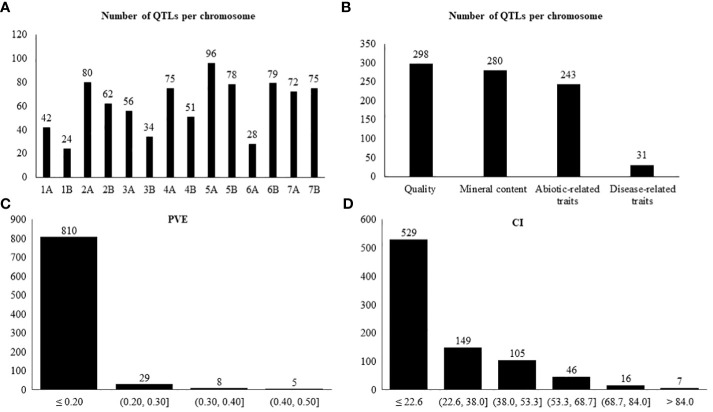
Number of QTLs per: **(A)** chromosome, **(B)** trait category, **(C)** PVE, **(D)** confidence interval based on 852 collected QTLs.

The 17 studies comprised 19 biparental populations and ten wild emmer accessions. In our study, only the QTLs belonging to the wild emmer parent were used. A total of 852 QTLs, distributed across all 14 chromosomes (A and B genomes) of wild emmer, for the 41 selected traits were collected (**Table 2**). The number of QTLs ranged from 24 on chromosome 1B to 96 on chromosome 5A, averaging 61 QTLs per chromosome (**Figure 1A**). Overall, quality was the category with the highest number of QTLs, representing 35% (298 QTLs), followed by mineral content with 33% (280 QTLs), abiotic-related traits with 28% (243 QTLs), and disease-related traits with 4% (31 QTLs) (**Figure 1B**). GPC was the trait with the largest number of QTLs (86), followed by TKW (58 QTLs) (**Table 1**).

The PVE for each QTL ranged from 0.001 to 0.63, with an average of 0.08 (**Figure 1C**). Per category, the PVE ranged as follows: abiotic-related traits from 0.001 to 0.46; disease-related traits from 0.02 to 0.26; mineral content from 0.005 to 0.24; and quality from 0.006 to 0.63 (**Supplementary Figure S1**). A total of 94%, 74%, 99%, and 95% of the QTLs for abiotic-related traits, disease-related traits, mineral content, and quality had PVE ≤ 0.20, respectively.

The CIs ranged from 0.1 to 234.5 cM, with an average of 22.6 cM. A total of 529 QTLs, corresponding to 62% of the studied QTLs, were located between 0.1 and 22.6 cM (**Figure 1D**). Of those 529 QTLs, 144 corresponded to abiotic-related traits, 23 to disease-related traits, 164 to mineral content, and 205 to quality (**Supplementary Figure S2**).

### 3.2 Projection of the QTLs on the consensus map

**Figure 2:**
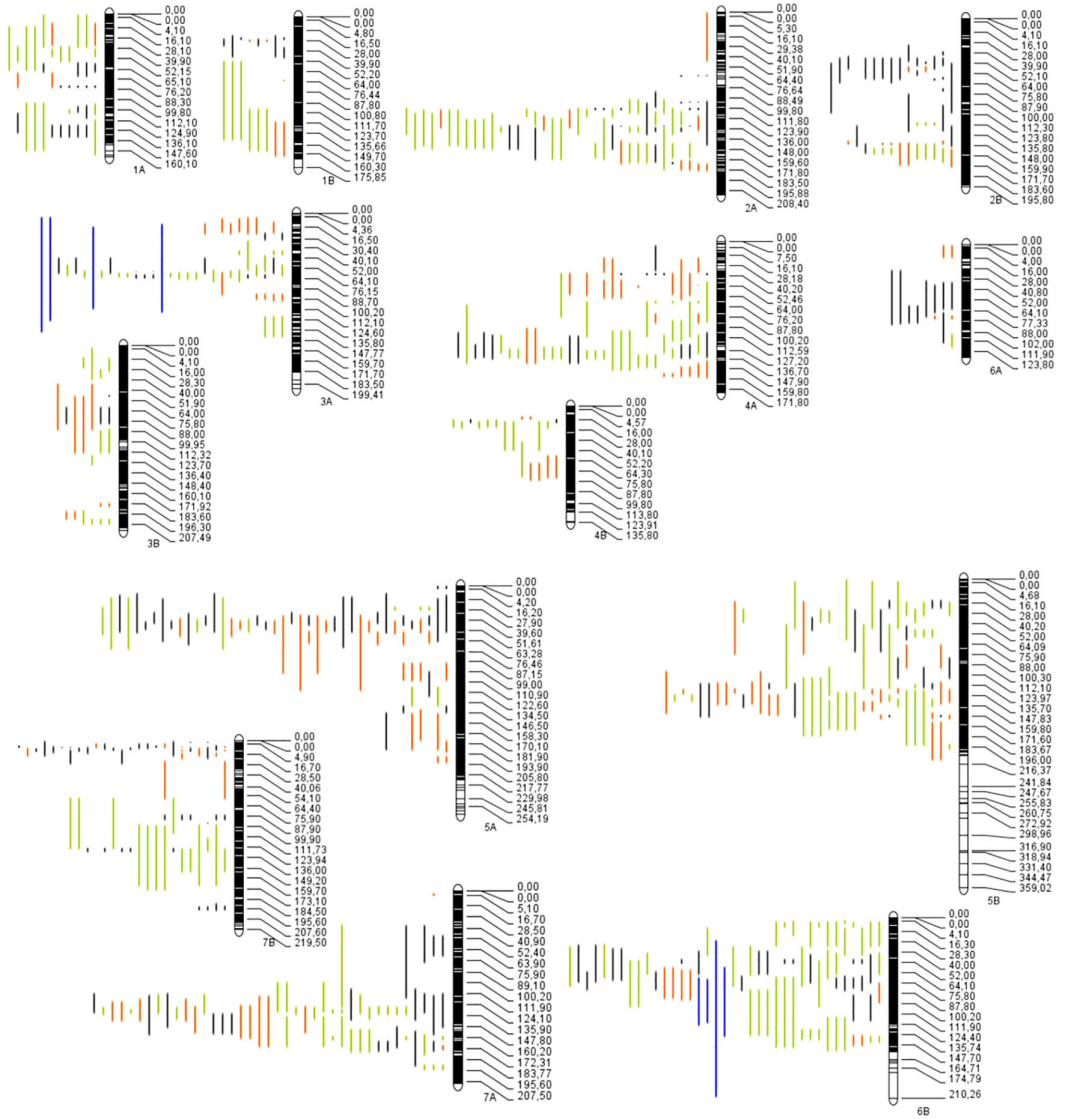
Distribution of the QTLs projected through 14 chromosomes in wild emmer for quality traits (red bars), mineral content (yellow bars), abiotic-related traits (green bars) and fusarium head blight (blue bars).

**Figure 3:**
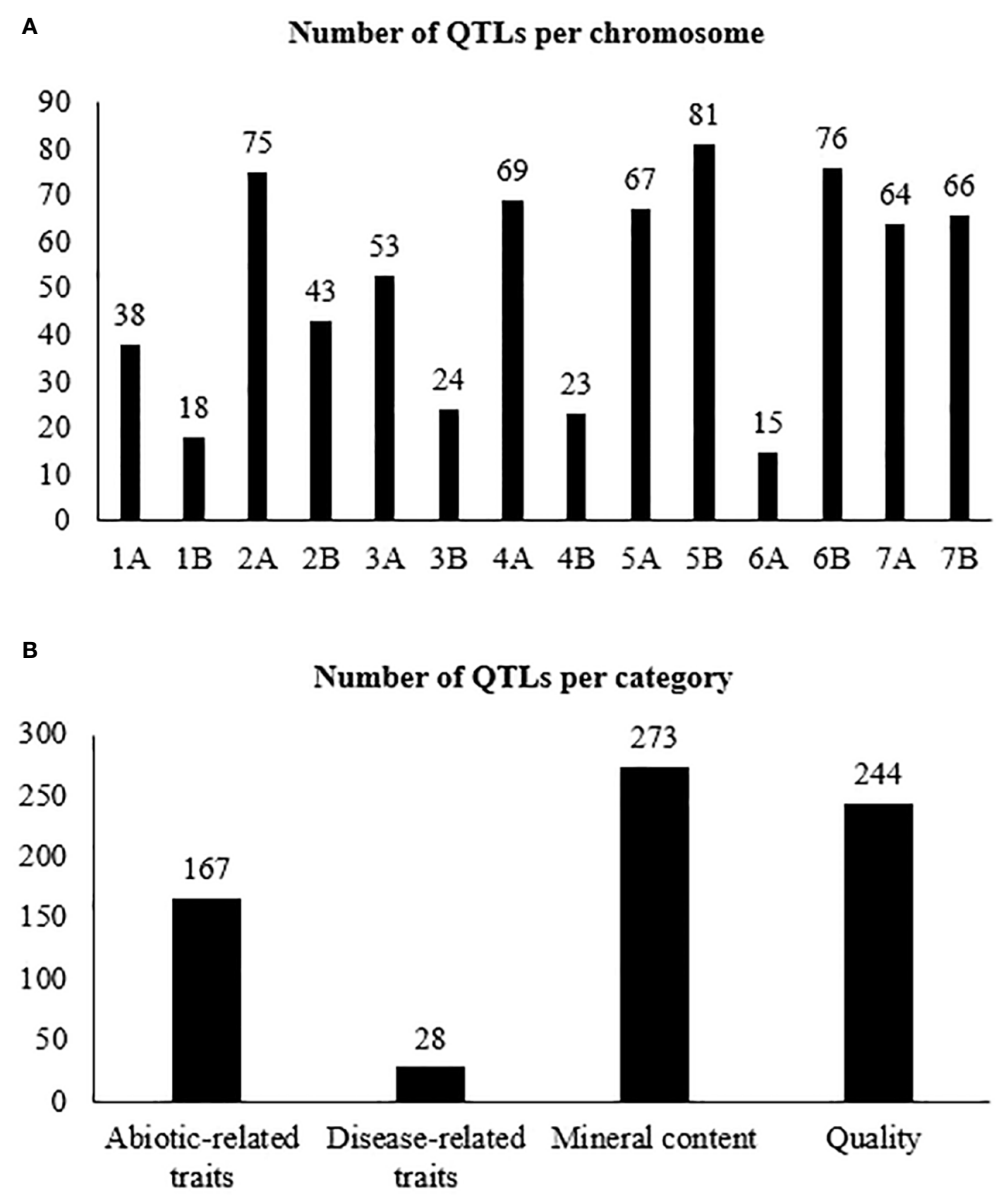
Number of projected QTLs per: **(A)** chromosome and **(B)** trait category.

The projection of the QTLs was made using the 17 studies collected previously indicated; however, only 14 studies were projected. Of the 852 QTLs available, only 712 were successfully projected on the consensus map (**Figure 2**); the remaining 140 QTLs could not be projected. According to Soriano and Alvaro (2019), the lack of projection is due to a low PVE between the QTLs, causing a larger CI and/or the absence of markers between the original map and the consensus map. The number of QTLs per chromosome ranged from 15 on chromosome 6A to 81 on chromosome 5B (**Figure 3A**). The trait with the most significant number of projected QTLs was GPC, with 85 QTLs representing 12% of the total QTLs. Plant height (PH), spike compactness (SC), and number of spikelets with one fertile floret (STPS) had the lower number of QTLs projected, with 1 QTL per trait, representing 0.14%, respectively (**Supplementary Table S2**). The category mineral content had the highest number of QTLs projected with 38%, followed by quality, abiotic-related traits, and disease-related traits with 34%, 23%, and 3.9%, respectively (**Figure 3B**).

The consensus map included 31,723 markers, covering a length of 2,879.1 cM. For each chromosome, the size of the genetic map ranged from 131.2 cM on chromosome 6A to 359.0 cM on chromosome 5B. In addition, the number of markers ranged from 1,455 in chromosome 4B to 3,421 in chromosome 2B, with an average of 2,266 marker per chromosome (**Supplementary Table S3**).

### 3.3 Meta-QTLs detected for all studied traits

Of the total 712 projected QTLs, only 395 QTLs were grouped into 43 MQTLs, which significantly reduced the total number of projected QTLs (43 MQTLs correspond to equal 11% of 395 QTLs). The remaining 317 QTLs could not be assigned to any MQTL, staying as a single QTL. This could happen because the predicted peaks were not included in any MQTL or the CI was too large. The number of MQTLs ranged from 2 in chromosomes 3B, 4A, 4B, 6A, and 7B to 5 in chromosomes 2A and 5A, with an average of 3.1 MQTLs per chromosome. *dicoccoides_MQTL_7A.2* was the MQTL with the highest number of QTLs associated (37 QTLs) vs. *dicoccoides_MQTL_1B.2*, *dicoccoides_MQTL_3B.2*, *dicoccoides_MQTL_5B.2*, *dicoccoides_MQTL_6A.2*, and *dicoccoides_MQTL_7A.1* with the lowest number of QTLs associated (3 QTLs).

Each MQTL was linked to at least one specific trait. For instance, *dicoccoides_MQTL_5A.1* and *dicoccoides_MQTL_7A.1* were associated with GPC and spike dry matter (SDM), respectively. Notably, *dicoccoides_MQTL_7A.2* exhibited associations with ten distinct traits, as detailed in **Table 3**.

**Table 3:** Characterization of the detected MQTLs for different traits in wild emmer.

MQTL	Chr	Position (cM)	CI (95%)	N QTLs	N studies	Left marker	Right marker	Traits
*dicoccoides_MQTL_1A.1*	1A	42.79	6.56	7	3	gwm111	IWA1803	GCaC, GFeC, FLRI
*dicoccoides_MQTL_1A.2*	1A	60.04	7.26	7	2	IWB53606	IWB48157	GPC, CIR
*dicoccoides_MQTL_1A.3*	1A	86.88	1.75	5	2	IWB9711	IWB279	G_P_C, TKW, GCaC, HI
*dicoccoides_MQTL_1A.4*	1A	114.91	5.34	6	2	IWA2404	IWB58389	GKC, TKW
*dicoccoides_MQTL_1B.1*	1B	28.16	1.99	6	2	barc119	IWA2577	DFPH, HI, KNPS
*dicoccoides_MQTL_1B.2*	1B	78.83	15.66	3	2	IWB35930	IWB7112	GFeC, TKW
*dicoccoides_MQTL_1B.3*	1B	135.28	28.75	5	2	IWA3497	IWB31755	GSeC, CHL
*dicoccoides_MQTL_2A.1*	2A	106.59	0.97	4	2	IWB7658	IWB8175	FHB, OPH
*dicoccoides_MQTL_2A.2*	2A	114.57	1.49	8	2	wPTt-8216	IWB16917	GMgC, GPC
*dicoccoides_MQTL_2A.3*	2A	139.34	3.68	5	2	IWB32349	IWB65467	TKW, GSC
*dicoccoides_MQTL_2A.4*	2A	161.03	9.35	7	3	IWA3629	SBG_188821	GKC, TKW
*dicoccoides_MQTL_2A.5*	2A	177.72	0.5	5	2	wPt-3865	IWB65261	FLRI, SHT
*dicoccoides_MQTL_2B.1*	2B	50.99	2.78	11	3	IWB71313	gwm429	HI, TKW,CL
*dicoccoides_MQTL_2B.2*	2B	123.8	2.89	5	3	IWA6453	IWB29225	GCuC, TKW, GPC
*dicoccoides_MQTL_2B.3*	2B	146.73	2.27	4	2	IWB2073	IWB41405	DHFM, DFPH
*dicoccoides_MQTL_2B.4*	2B	156.7	1.52	8	2	IWA8449	IWA3478	GFeC, GMgC, GZnC, FLW
*dicoccoides_MQTL_3A.1*	3A	50.3	0.95	9	3	SBG_138668	IWA2898	GSC, PH, TKW
*dicoccoides_MQTL_3A.2*	3A	74.35	2.31	10	3	IWA5164	IWB69857	GMgC, GMnC, GPC
*dicoccoides_MQTL_3A.3*	3A	100.25	1.54	7	2	IWB35484	IWB73344	CIR, GSC
*dicoccoides_MQTL_3B.1*	3B	85.86	10.46	9	2	IWB40525	IWB52023	CL, OPH, GPC, GZnC, GMnC
*dicoccoides_MQTL_3B.2*	3B	198.7	8	3	2	IWB71781	IWB10839	FLW, GFeC
*dicoccoides_MQTL_4A.1*	4A	38.36	1.65	9	2	IWA4321	IWB34953	CHL, HI, GPC
*dicoccoides_MQTL_4A.2*	4A	131.23	3.75	31	4	IWB14913	wmc718	GCaC, GCuC, GPC, G_P_C, GSC, SDM, DFHM, GZnC
*dicoccoides_MQTL_4B.1*	4B	16.38	3.14	13	2	IWB63596	WB34526	GFeC, GPC, GKC, CHL, GCuC
*dicoccoides_MQTL_4B.2*	4B	78.98	10.14	5	2	IWA6197	IWB32903	DFPH, FLL, G_P_C
*dicoccoides_MQTL_5A.1*	5A	35.99	5.11	8	2	IWB31441	IWB30321	GPC
*dicoccoides_MQTL_5A.2*	5A	48.93	3.65	18	2	IWB59189	IWB14724	DFPH, FLL, GFeC, SL, CL, FLRI
*dicoccoides_MQTL_5A.3*	5A	106.33	11.09	5	2	IWA6573	WB2066	CIR, TKW
*dicoccoides_MQTL_5A.4*	5A	142.55	7.03	5	3	IWB3132	SBG_143269	CL, GPC, GSC, HI
dicoccoides_MQTL_5A.5*	5A	190.41	0.01	6	2	IWA7162	IWB12799	CIR, DFHM, SC
*dicoccoides_MQTL_5B.1*	5B	52.54	0.23	25	7	IWA7227	IWA5280	GCaC, GCuC, GSC, GSeC, GPC, HKW, TKW, STSP, FLRI
*dicoccoides_MQTL_5B.2*	5B	123.74	4.8	3	2	IWB40925	barc337	GSC, HI
*dicoccoides_MQTL_5B.3*	5B	142.43	4.11	11	2	IWB63594	kbo_0309	FLW, CHL, GSC, GMgC, DFHM
*dicoccoides_MQTL_6A.1*	6A	59.32	0.69	5	3	IWB51739	IWB66638	FHB, TKW
*dicoccoides_MQTL_6A.2*	6A	84.95	4.31	3	2	IWB9445	IWB4417	CHL, GPC
*dicoccoides_MQTL_6B.1*	6B	52.19	3.48	12	2	gwm518	barc68	GAlC, GPC, GSC
*dicoccoides_MQTL_6B.2*	6B	74.40	0.17	6	3	IWA5966	IWB56000	CIR, CL, FHB, GPC, HI
*dicoccoides_MQTL_6B.3*	6B	140.58	1.51	9	3	IWB62788	IWB8526	CHL, GCaC, GFeC, GMgC
*dicoccoides_MQTL_7A.1*	7A	59.6	16.59	3	2	SBG_116368	IWA472	SDM
*dicoccoides_MQTL_7A.2*	7A	132.57	2.5	37	5	IWA7089	IWB45735	CHL, CL, FLW, GCuC, GFeC, GSC, GZnC, GPC, SL, TKW
*dicoccoides_MQTL_7A.3*	7A	170.72	5.48	4	2	IWB39743	wPt-5558	GPC, DFHM
*dicoccoides_MQTL_7B.1*	7B	8.5	0.59	17	2	IWB26957	IWB71139	CL, DFHM, GY, KNPS, HI, TKW, SDM
*dicoccoides_MQTL_7B.2*	7B	95.98	1.15	10	4	IWA449	IWB56558	GSeC, GZnC, SL, TKW
*dicoccoides_MQTL_7B.3*	7B	129.11	3.02	15	3	IWB23685	IWB24797	GPC, GMgC, GSC

*The flanking markers, while being the closest ones to dicoccoides_MQTL_5A.5, were situated outside the defined boundaries of the MQTL; Chr, chromosome; CI, confidence interval; N, number.

In the overall analysis, three of the 43 MQTLs were identified with associations to FHB on chromosomes 2A, 6A, and 6B, specifically *dicoccoides_MQTL_2A.1*, *dicoccoides_MQTL_6A.1*, and *dicoccoides_MQTL_6B.2*. Furthermore, a subset of four MQTLs out of the total 43 were exclusively associated with quality traits, distributed across chromosomes 2A, 5A, and 7A. Examples include *dicoccoides_MQTL_2A.4*, *dicoccoides_MQTL_2A.3*, *dicoccoides_MQTL_5A.1*, and *dicoccoides_MQTL_7A.1*. Similarly, two MQTLs, specifically *dicoccoides_MQTL_2B.3* and *dicoccoides_MQTL_5A.5*, associated solely with abiotic-related traits within chromosomes 2B and 5A. Notably, 26 out of the 43 MQTLs associated with mineral content and they also showed connections to one or more quality traits and abiotic-related traits, as outlined in **Table 3**.

The detected MQTLs exhibited a range of average CI (95%), from 0.01 cM for *dicoccoides_MQTL_5A.5* to 28.75 cM for *dicoccoides_MQTL_1B.3*, with an overall mean of 4.78 cM (as shown in **Table 3**). Interestingly, this average was 4.7 times smaller than the initial average CI of 22.6 cM. The genomic region delineated for *dicoccoides_MQTL_5A.5* (CI: 0.01 cM) exhibited an association with six QTLs originating from distinct studies, one conducted by Buerstmayr et al. (2012) and the other by Fatiukha et al. (2021), as well as two wild emmer accessions (**Table 2**). Notably, these QTLs exclusively pertained to traits categorized as abiotic related, specifically the carbon isotope ratio (CIR), days from heading to maturity (DFHM), and SC. It is noteworthy that the flanking markers associated with these QTLs were situated external to the defined MQTL region (**Table 3**). No discernible genetic associations with genes were identified in proximity to these markers.

Per chromosome, the average CI ranged from 1.57 cM in chromosome 7B to 15.47 cM in chromosome 1B.

### 3.4 Candidate genes

Using the genomic sequences located in the breeding QTL from “Svevo,” nine candidate genes correlated to MQTL were identified (**Table 4**). The flanking markers were aligned with the genome browsers for both Svevo (durum wheat) reference genome, accessible at https://iwgs.org/, and after excluding transposable elements, a total of 25 genes were identified.

**Table 4:** Selected candidate genes per MQTL.

MQTL	Chr	Position (cM)	Traits	Genes
*dicoccoides_MQTL_1A.1*	1A	42.79	GCaC, GFeC, FLRI	*beta-1,3-galactosyltransferase*
*dicoccoides_MQTL_1A.2*	1A	60.04	GPC, CIR	*glycosyltransferase STELLO2*
*dicoccoides_MQTL_1A.4*	1A	114.91	GKC, TKW	*SUPPRESSOR OF PHYA-105 1-like*
*dicoccoides_MQTL_2A.4*	2A	161.03	GKC, TKW	*kinesin-like protein KIN-14I*
*dicoccoides_MQTL_2A.5*	2A	177.72	FLRI, SHT	*cysteine-rich receptor-like protein kinase 10*
*dicoccoides_MQTL_4A.1*	4A	38.36	CHL, HI, GPC	*alpha-L-fucosidase 2-like*
*dicoccoides_MQTL_4B.1*	4B	16.38	GFeC, GPC, GKC, CHL, GCuC	*aminopeptidase M1-B*
*dicoccoides_MQTL_6B.2*	6B	74.4	CIR, CL, FHB, GPC, HI	*glucose-6-phosphate 1-dehydrogenase*
*dicoccoides_MQTL_7B.3*	7B	129.11	GPC, GMgC, GSC	*xyloglucan endotransglucosylase/hydrolase protein 24-like*

Chr, chromosome.

The MQTLs that were associated with candidate genes were located on chromosomes 1A, 2A, 4A, 4B, 6B, and 7B (**Table 4**). In the case of chromosome 1A, three MQTLs were associated with genes. In detail, *dicoccoides_MQTL_1A.1* was associated with *beta-1,3-galactosyltransferase*, *dicoccoides_MQTL_1A.2* was associated with *glycosyltransferase* STELLO2, and *dicoccoides_MQTL_1A.4* was associated with *SUPPRESSOR OF PHYA-105 1-like*. The chromosome has associated the *dicoccoides_MQTL_2A.4* with the *kinesin-like protein KIN-14I* and the *dicoccoides_MQTL_2A.5 cysteine-rich receptor-like protein kinase* (*CRK*) *10*. The MTQL for chromosomes 4A, 4B, 6B, and 7B were associated with one gene each.

## 4 Discussion

### 4.1 The significance of considering MQTL analysis

In recent years, various studies have been conducted on modern wheats, particularly bread wheat, to identify new genetic locations associated with different quality parameters. These parameters include yield and related traits (Maccaferri et al., 2019; Arriagada et al., 2020; Gupta et al., 2020), protein content (Saini et al., 2022c), mineral content (Fatiukha et al., 2020), abiotic-related traits (Bhusal et al., 2018), and biotic-related traits such as FHB (Zhang et al., 2022). Nevertheless, research indicates that intensive breeding practices in modern wheats have led to increased productivity at the expense of diminished nutritional value, protein content, and resistance or tolerance to abiotic and biotic stressors (Cakmak et al., 2010; Peng et al., 2011; Cabas-Lühmann et al., 2023). Various QTL studies were conducted, showing the substantial nutritive value and stress tolerance conferred by wild emmer. These studies primarily aimed to identify alleles or markers associated with nutritional value, particularly concerning iron and zinc content (Peleg et al., 2009a; Liu et al., 2021), protein content (Fatiukha et al., 2019), and tolerance or resistance to abiotic (Peleg et al., 2005) and biotic stressors (Huang et al., 2016). Some of these QTLs have been used in wheat breeding programs to enhance the genotypes by introducing alleles that broaden their genetic diversity, thereby improving nutritional content, protein content, and resistance or tolerance to stressors (Kumar et al., 2018; Fatiukha et al., 2019; Kuznetsova et al., 2019; Cabas-Lühmann et al., 2023). However, despite all these studies on QTLs, wheat breeding programs have used only a limited subset of those genetic resources (Cobb et al., 2019). This limited adoption is primarily because the expression of these QTLs is greatly affected by the environment, the genotype by environment interactions, the mapping population type, and the statistical method used for the QTL identification (Sandhu et al., 2021; Zheng et al., 2021). A Meta-QTL analysis emerges as a powerful approach to consolidate QTL information from diverse populations cultivated in various environments. This method identifies stable QTLs within the plant genome, situated in genomic regions encompassing valuable and diverse genetic material for potential integration into wheat breeding programs.

### 4.2 Quantitative trait loci for quality traits, mineral content, abiotic-related traits, and disease-related traits

In this study, a total of 852 QTLs were identified across all 14 chromosomes of wild emmer. The QTLs were distributed as quality traits (298 QTLs), mineral content (280 QTLs), abiotic-related traits (243 QTLs), and disease-related traits (31 QTLs). The quality category exhibited the highest QTL count, encompassing yield, yield-related traits, and GPC. Within the quality category, TKW and GPC were the traits with the highest QTL counts, having 58 and 89 QTLs, respectively. The largest number of QTLs associated with TKW and GPC can be linked to the extensive research conducted on these traits, given their significance in both wheat yield and product manufacturing (Avni et al., 2018). For instance, QTL studies on yield in durum wheat often utilize TKW as a pivotal yield component (Liang et al., 2017), influenced by environmental factors during grain filling (Li et al., 2019), that can be used for genotypes that grow under diverse environmental conditions (Gupta et al., 2020; Arriagada et al., 2022; Wang et al., 2022). Since the obtention of high-yield genotypes is necessary for wheat breeding programs, incorporating accumulated loci associated with yield components like TKW becomes essential. TKW is not only influenced by the environment during grain filling, but it is also positively correlated with overall grain and milling yield (Wang and Fu, 2020). On the other hand, wild emmer is known as a valuable genetic reservoir, abundant in allelic variants, offering a substantial alternative to enhance GPC through the introgression of favorable alleles into modern wheat varieties (Fatiukha et al., 2019; Colasuonno et al., 2021). This explains why most of the QTL studies on this ancient wheat are related to GPC. Interestingly, the data highlights that the primary locations for QTLs related to TKW were chromosomes 5A (13 QTLs) and 4A (11 QTLs). In contrast, QTLs for GPC were predominantly localized on chromosomes 6A and 7B (8 QTLs each).

Mineral content was the second-highest category in the number of QTLs, which can be attributed to the substantial genetic diversity inherent in mineral nutrient concentrations associated with wild emmer (Cakmak et al., 2004; Peleg et al., 2008). A known QTL called *Gpc-B1* has enhanced both GPC and mineral content upon introgression into modern wheat. Initially identified by Joppa and Cantrell (1990) on chromosome 6B in wild emmer lines FA-15-3 and F-28-8-3 from Israel, *Gpc-B1* has proven its efficacy. Research by Distelfeld et al. (2007) has shown that recombinant chromosome substitution lines (RSLs) carrying the wild *Gpc-B1* allele from the cross “DIC-6B” x LDN exhibited, on average, 12%, 18%, and 29% higher concentrations of Zn, Fe, and Mn, respectively, in the grain compared to LDN. Furthermore, Peleg et al. (2009a) identified 38 stable QTLs associated with the wild alleles from G18-16 within a RIL population. These QTLs explained variations in grain mineral nutrient concentrations ranging from 0.7% to 19.2%. In the current study, most of the collected QTLs related to mineral content were located on chromosomes 6B (40 QTLs), 2A (34 QTLs), and 7A (32 QTLs).

Abiotic-related traits comprised the next category, followed by disease-related traits with only FHB as a trait of interest. The number of QTLs associated with abiotic-related traits is due to the focus of many studies on identifying genome locations that confer tolerance to drought conditions because of the global warming impact on crop productivity (Shew et al., 2020). Wild emmer can grow under dry and saline conditions and it has promising genes allowing to cope with these environmental constraints (Peng et al., 2003). According to our data, most of the QTLs for abiotic-related traits were on chromosomes 5A (35 QTLs) and 5B (33 QTLs). Peleg et al. (2009b) identified 59 QTLs in the wild emmer accession G18-16 related to better adaptability of the crop grown under drought conditions from an original RIL population (durum LDN × G18-16). The major genomic regions associated with productivity and drought-adaptability traits were identified on chromosomes 2A, 4A, 5A, and 7B.

FHB is a severe fungal disease-causing significant yield losses, quality deterioration, and mycotoxin contamination in small grains, particularly in crops like wheat, with a pronounced impact on durum wheat. Breeding for FHB resistance poses challenges due to the polygenic nature of the trait and the substantial influence of genotype by environment interaction. Burstmayr et al. (2020) underscore the importance of considering genetic variation and employing appropriate tools for identifying genotypes for this trait in the wheat breeding process. Two distinct types of resistance have been identified: Type 1, which addresses initial infection resistance, and Type 2, which is the resistance of the spread within the spike. Type 2 resistance is considered less susceptible to environmental variations and a more reliable indicator of FHB resistance (Buerstmayr et al., 2013). Wild emmer QTLs have been related to both FHB resistance (Otto et al., 2002; Chen et al., 2007; Gladysz et al., 2007; Buerstmayr et al., 2013) and susceptibility (Garvin et al., 2009), serving as a potential resource for enhancing durum wheat genotypes.

Our study showed that the majority of FHB QTLs were situated on chromosome 2A (15 QTLs), followed by chromosome 6B (six QTLs), and chromosome 3A (four QTLs). On chromosome 3A, the QTL *Qfhs.ndsu-3AS* derived from wild emmer “Israel A” (Otto et al., 2002; Chen et al., 2007), “Mt. Hermon” (Gladysz et al., 2007), and “Mt. Gerizim #36” (Buerstmayr et al., 2013) imparted Type 2 resistance. All three accessions exhibited a peak position near the *Xgwm2 microsatellie marker*. Soresi et al. (2017) validated the effectiveness of *Qfhs.ndsu-3AS* resistance in two Argentinean durum wheat cultivars. *Qfhs.ndsu-3A* demonstrated a dominant allele interaction, enhancing resistance by 50% for both homozygous and heterozygous genotypes.

Garvin et al. (2009) identified specific QTLs on the long arm of chromosome 2A within the wild emmer accession “Israel A,” precisely located between *Xgwm558* and *Xgwm445*, covering an approximate distance of 22 cM. Their findings suggested that chromosome 2A contributes to increased susceptibility to FHB. One hypothesis proposed by those researchers was that the genes present on chromosome 2A might mitigate or suppress the effect of FHB resistance conditioned by *Qfhs.ndsu 3AS*. In a related study, Faris et al. (2014), exploring the association between spike morphology and FHB in wild emmer, reported a QTL for spikelet numbers per spike (*QSpn.fcu-2A*) that overlapped with the CI of the QTL for FHB susceptibility described by Garvin et al. (2009) on chromosome 2A. It was noted, however, that although they shared the same genomic region, the QTL for spikelet numbers per spike (*QSpn.fcu-2A*) was excluded as a determinant of FHB susceptibility due to its consideration as an attribute of FHB resistance.

### 4.3 Meta-QTL and for quality traits, mineral content, abiotic-related traits, and disease-related traits

In this study, we conducted the first MQTL analysis for multiple polygenic traits in wild emmer, and we grouped a total of 395 previously identified QTLs into 43 MQTLs. The results demonstrated a reduction of existing QTL information for polygenic traits, aligning with findings by Khahani et al. (2021), indicating that MQTL analysis enhances precision in detecting candidate genes by narrowing down the genomic regions for traits of interest. The average CI of the MQTLs was much narrower than the average CI of the known QTLs. Specifically, the average CI of the MQTLs was 4.7-fold lower than the initial average CI (22.6 cM). This reduction in CI means increased accuracy and facilitates a more focused exploration of promising candidate genes within each MQTL (Sandhu et al., 2021). This refined approach provides a valuable resource for further allele introgression in wheat breeding.

The MQTL, specifically *dicoccoides_MQTL_7A.2*, demonstrated clear strength by encompassing the largest number of associated QTLs (37) from diverse environmental backgrounds. In general, only a limited number of MQTLs were specifically linked to a particular category; for quality, there were four of 43 MQTLs, and for abiotic-related traits, two of 43 MQTLs exhibited category specificity. The remaining 37 MQTLs exhibited a polygenic trait from various categories. Interestingly, all MQTLs involving mineral content traits were associated with traits from other categories, primarily quality and abiotic-related traits. This information holds significance for wheat breeding programs, as the identified MQTLs were linked to multiple polygenic traits. This versatility is important for potential enhancements in productivity and overall quality, addressing key and challenging traits in wheat breeding.

Previous MQTL studies in durum wheat have shown similar co-localization compared to the ones generated in this research. Nine MQTLs found by Soriano et al. (2021) were closer to or overlapped the positions of the ones found in this study in chromosomes 1A (one MQTL; peak 46.1 cM), 1B (one MQTL; peak 29.3 cM), 2B (two MQTLs; peaks 59.0 and 147.5 cM), 4B (one MQTL; peak 17.1 cM), 5A (two MQTLs; peaks 48.6 and 143.2 cM), and 5B (two MQTLs; peaks 122.2 and 139.0 cM). Ten MTQLs identified by Arriagada et al. (2022) were closer to or overlapped the positions of the ones encountered in this research in chromosomes 1A (one MQTL; peak 119.2 cM), 1B (one MQTL; peak 71.5 cM), 2A (two MQTLs; peak 139.1 and 139.3 cM), 2B (two MQTLs; peak 56.1 and 51.04 cM), 3A (one MQTL; peak 75.8 cM), 5A (two MTQL; peak 36.6 cM and 102.83), 5B (one MQTL; peak 44.5 cM), and 6B (one MQTL; peak 74.1 cM). Arriagada et al. (2022) identified some of these MQTLs under rainfed and/or irrigated conditions of durum wheat. Except for the MQTL found in chromosomes 2A (one MQTL), 2B (one MTQL), 3A (one MQTL), and 5B (one MTQL), the rest were located under rainfed conditions, and all of them related to yield traits. One genomic region in chromosome 2B was similar and closer in all three studies reported by Soriano et al. (2021), *durumMQTL2B.2* (peak 59.0 cM) that was associated with grain selenium yield and GPC; by Arriagada et al. (2022), *yield_MQTL2B.1_I* (peak 56.12 cM) that was associated with TKW and HI; and in the present study, *dicoccoides_MQTL_2B.1* (peak 50.99 cM), which associated with HI, GY, and CL. Although no similar genes were identified in this genomic region, the MQTLs with abiotic and yield-related traits under adverse growing conditions can be considered strong genomic regions for wheat breeding programs to maintain or increase yield under drought conditions, being a powerful resource in the current worldwide scenario of climate change.

### 4.4 Candidate genes identified for the stable MQTLs

Since this is the first work that tackles multiple traits in wild emmer, the obtained genes of interest are valuable for their study and potential use by utilizing the durum wheat reference genome.

In particular, on chromosome 1A, the *MQTL_1A.1* for GCaC, GFeC, and FLRI was associated with a *beta-1,3-galactosyltransferase* gene involved in the transfer of a galactose to a terminal *GlcNAc* residue in β-1,3-linkage (Rathan et al., 2022). Another association with genes involved in the cell wall composition, the *glycosyltransferase STELLO2*, was detected again on chromosome 1A associated with *MQTL_1A.2* (Gómez-Espejo et al., 2022). On chromosome 1A, a gene was identified, the suppressor of the *PHYTOCHROME A* (PHYA), which is involved in regulating mature plant development and related to TKW and GKC (Garg et al., 2006). Another correlation with TKW and GKC was reported with MQTL on chromosome 2A and the gene *kinesin-like protein KIN-14l*, which regulates grain length and PH by affecting expression levels of genes involved in GA synthesis and response (Wu et al., 2014). An important association was detected on chromosome 2A with the CRK and FLRI abiotic-related trait. Many studies suggest the involvement of CRK proteins in plant development, cell death, immunity, and responses to abiotic stresses (Shumayla et al., 2019). A gene related to carbohydrate metabolism, the *alpha-L-fucosidase 2-like* was detected on chromosome 4A and co-localized with MQTL for wheat quality. The literature reported the importance of this gene in influencing floral organ differentiation, cell wall biogenesis and degradation, and spike differentiation (Zhu et al., 2016). A BLAST search revealed an association with a candidate gene, *aminopeptidase M1-B*, involved in plant developmental processes such as embryonic, vegetative, and reproductive development in agreement with Maulana et al. (2021) in wheat.

Another relevant association was detected on chromosome 6B between a MQTL controlling different traits and the *glucose-6-phosphate 1-dehydrogenase* gene, which is involved in regulating the oxidative pentose phosphate pathway. This gene plays a central role during nitrate assimilation in heterotrophic tissues, contributing to nitrogen metabolism (Esposito, 2016).

The last association was reported on chromosome 7B between the *xyloglucan endotransglucosylase/hydrolase protein 24-like* gene and GPC, GMgC, and GSC. The *xyloglucan endoglycosylases/hydrolases* proteins are involved in constructing and remodeling cell wall structures and play an essential role in regulating cell wall extensibility (Han et al., 2023).

## 5 Conclusions

This comprehensive study was the first work that analyzed 852 already known QTLs across 19 biparental populations of wild emmer, shedding light on the genetic basis of 41 selected traits. The traits were reported according to their category as quality traits, mineral content, abiotic-related traits, and disease-related traits. These QTLs were distributed across all 14 chromosomes and exhibited a wide range of phenotypic variation (0.001 to 0.63), with GPC and TKW emerging as the traits with more QTLs reported in the literature.

Overall, 43 MQTLs were determined, providing a condensed view of stable genomic regions associated with polygenic traits. These MQTLs exhibited narrower CIs than known QTLs, with an average of 4.7-folds lower. The *dicoccoides_MQTL_7A.2* had the most QTLs associated with a total of 37 QTLs from different environmental backgrounds, reflecting the strength of the found MQTLs. On the other hand, there was a genomic region, the *dicoccoides_MQTL_2B.1* in chromosome 2B, similar to two other studies: Soriano et al. (2021), *durumMQTL2B.2* (peak 59.0 cM) and Arriagada et al. (2022), *yield_MQTL2B.1_I* (peak 56.12 cM). This offers valuable insights for wheat breeding programs since MTQLs with abiotic and yield-related traits can be considered strong genomic regions to maintain or increase yield under stressful conditions. The obtained MQTLs represent regions rich in candidate genes, including those related to carbohydrate metabolism, plant development, and stress responses. This research underscores the significance of MQTL analysis in harnessing the genetic potential of wild emmer to enhance wheat productivity and quality in the face of evolving sustainable agricultural challenges and climate change.

## Data availability statement

The original contributions presented in the study are included in the article/**Supplementary Material**. Further inquiries can be directed to the corresponding authors.

## Author contributions

PC: Conceptualization, Data curation, Formal Analysis, Investigation, Writing – original draft, Writing – review & editing. AS: Conceptualization, Funding acquisition, Supervision, Writing – review & editing. OA: Data curation, Formal Analysis, Writing – review & editing. IvM: Writing – review & editing. IM: Writing – review & editing. CA: Writing – review & editing. AG: Writing – review & editing.
