# Supplementary figures and images for: Meta-QTL analysis and candidate genes for quality traits, mineral content, and abiotic-related traits in wild emmer
Source: Front Plant Sci. 2024 Mar 14;15:1305196. doi: 10.3389/fpls.2024.1305196 (PMC10972855; doi:10.3389/fpls.2024.1305196)

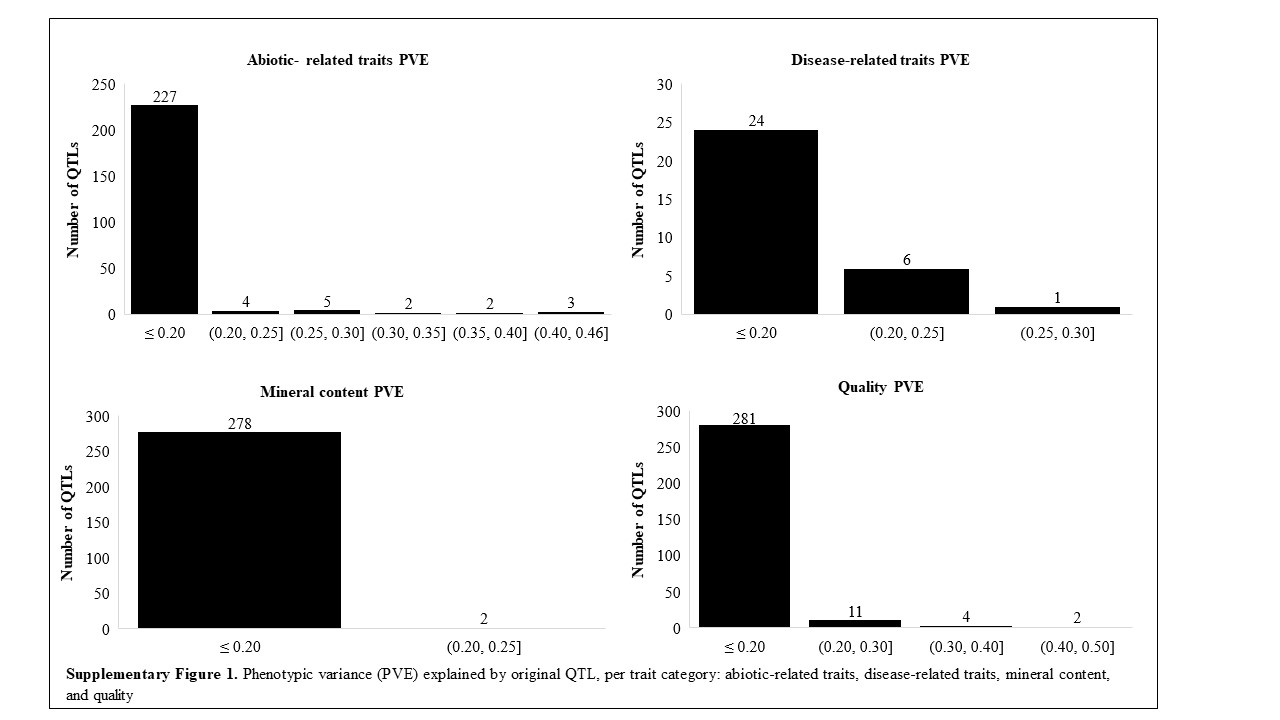

Supplement: Supplementary file 1 [file Image_1.jpg]

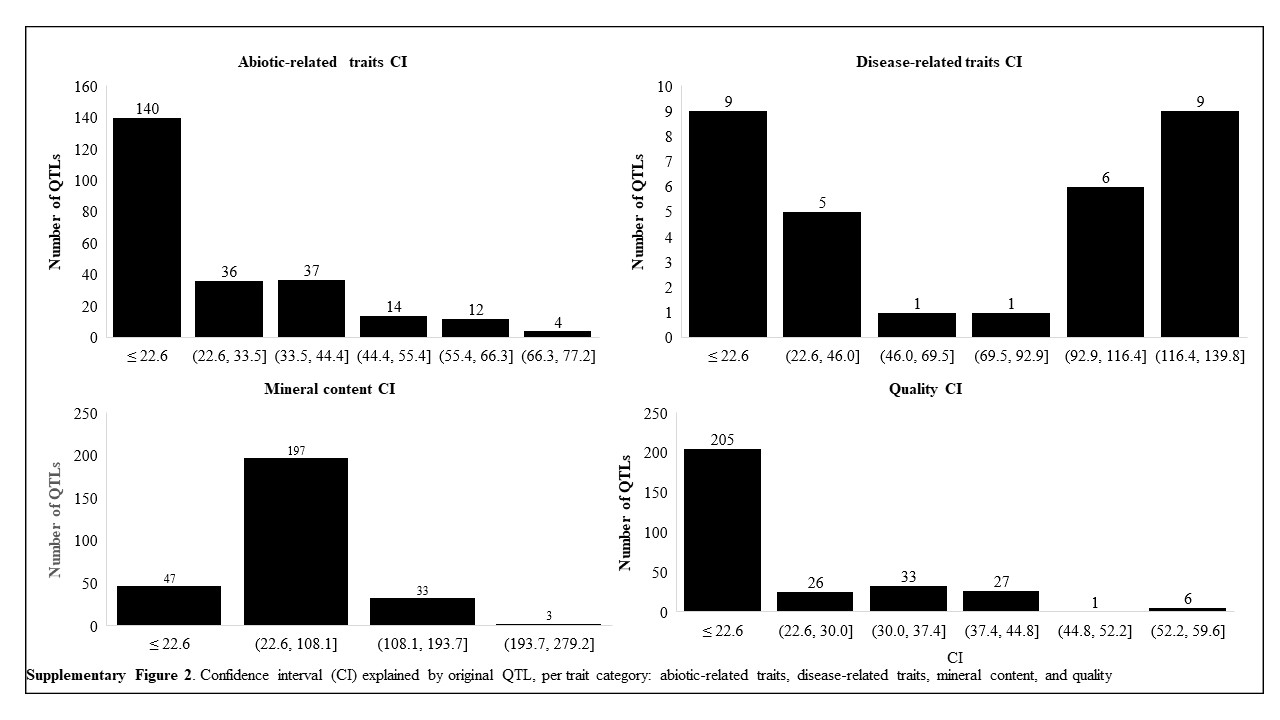

Supplement: Supplementary file 2 [file Image_2.jpg]
